# A comparison between electromechanical and pneumatic-controlled knee simulators for the investigation of wear of total knee replacements

**DOI:** 10.1177/0954411917696519

**Published:** 2017-03-25

**Authors:** Abdellatif Abdelgaied, John Fisher, Louise M Jennings

**Affiliations:** Institute of Medical and Biological Engineering, University of Leeds, Leeds, UK

**Keywords:** Knee replacement, electromechanical simulator, pneumatic simulator, polyethylene, wear

## Abstract

More robust preclinical experimental wear simulation methods are required in order to simulate a wider range of activities, observed in different patient populations such as younger more active patients, as well as to fully meet and be capable of going well beyond the existing requirements of the relevant international standards. A new six-station electromechanically driven simulator (Simulation Solutions, UK) with five fully independently controlled axes of articulation for each station, capable of replicating deep knee bending as well as other adverse conditions, which can be operated in either force or displacement control with improved input kinematic following, has been developed to meet these requirements. This study investigated the wear of a fixed-bearing total knee replacement using this electromechanically driven fully independent knee simulator and compared it to previous data from a predominantly pneumatically controlled simulator in which each station was not fully independently controlled. In addition, the kinematic performance and the repeatability of the simulators have been investigated and compared to the international standard requirements. The wear rates from the electromechanical and pneumatic knee simulators were not significantly different, with wear rates of 2.6 ± 0.9 and 2.7 ± 0.9 mm^3^/million cycles (MC; mean ± 95% confidence interval, p = 0.99) and 5.4 ± 1.4 and 6.7 ± 1.5 mm^3^/MC (mean ± 95 confidence interval, p = 0.54) from the electromechanical and pneumatic simulators under intermediate levels (maximum 5 mm) and high levels (maximum 10 mm) of anterior–posterior displacements, respectively. However, the output kinematic profiles of the control system, which drive the motion of the simulator, followed the input kinematic profiles more closely on the electromechanical simulator than the pneumatic simulator. In addition, the electromechanical simulator was capable of following kinematic and loading input cycles within the tolerances of the international standard requirements (ISO 14243-3). The new-generation electromechanical knee simulator with fully independent control has the potential to be used for a much wider range of kinematic conditions, including high-flexion and other severe conditions, due to its improved capability and performance in comparison to the previously used pneumatic-controlled simulators.

## Introduction

Surface wear of polyethylene and the resulting osteolysis are still considered a long-term risk factor of total knee replacements (TKRs), particularly as life expectancy and activity levels increase.^1^ Failed TKRs require expensive revision surgery, associated with a higher risk of post-surgery complications than the primary surgery.^2–6^ The number of recorded TKR revisions in 2014 in the United Kingdom was 3239.^7^ Unsurprisingly, the revision rate for young patients (under 55 years) was 10 times that for patients over 75 years.^7^ The current international standard for preclinical knee wear simulation has been based on the average patient and walking gait activity.^8,9^ In an attempt to address and understand the higher failure rates reported for young patients, preclinical testing methods which include a wider range of physiological conditions have also been developed.^10–13^

Experimental wear simulation is an established method for evaluating the wear performance of total joint replacements, with numerous publications over the last decade demonstrating the influence of design, material, kinematics, and sterilisation processes on the performance of TKRs.^2,10,14–18^ The average patient and walking gait activity have also been the basis for the development of experimental wear simulators.^19–22^ Although this has been effective at evaluating designs that have a lower average wear rate, such as cross-linked polyethylene,^15,23^ it does not replicate the variation and spread of in vivo wear rates. To further understand, evaluate, and reduce failure rates, it is necessary to use adverse conditions in in vitro simulations.^10,24^

The first-generation ProSim knee simulator (Manchester, UK) has been used over the last 15 years to determine the wear of knee prostheses.^1,14,15,17,23,25–29^ This six-station simulator had 6 degrees of freedom, with four controlled axes of motion, and was predominantly pneumatically controlled (three out of the four axes). The fourth axis, flexion/extension (FE), was electromechanically driven, with every three stations driven by one motor. The lack of fully independent control was a limitation of the simulator.^25,26,30^ The simulator also allowed passive abduction–adduction (AA) and a medial–lateral (ML) offset to be predefined. The anterior–posterior (AP) displacement and the internal–external (IE) rotation could be controlled using either force or displacement inputs, while the axial load was force controlled. The simulator was capable of reproducing the defined kinematic inputs within the limits of ± 1 mm in the AP and ± 0.7° in the IE (95% confidence intervals (CIs)), when running with unconstrained geometry (flat inserts). The simulator capability was, however, reduced when running with more constrained knee prostheses such as PFC Sigma (DePuy Synthes, UK) and when the output kinematics delivered by the control system to the prostheses could not precisely follow the input profiles due to the compressible pneumatic nature of the simulator^25^ and response to the resistance and inertia in the devices. Moreover, it was difficult to control the simulator air system in terms of positioning, accuracy, and repeatability. In addition, the simulator showed higher inter-station variability in terms of input kinematic profile following.^15,25,30^ Such variability could influence the ability to differentiate wear rates between different devices and conditions.

Knee wear simulators can often be driven using either displacement or force-controlled conditions for the AP displacement and IE rotation of the tibial component. There has been much debate whether displacement or force control regimes should be used.^25,31–35^ The displacement-controlled conditions allow for a well-defined and controlled number of variables, which can easily be implemented and can therefore reliably answer specific preclinical research questions. However, the displacement control test conditions use pre-specified kinematics for AP displacement and IE and do not, therefore, account for the effects of TKR design parameters (such as the conformity between the articulating surfaces) on the AP and IE motions. The displacement control conditions may not therefore necessarily reflect the in vivo conditions, where the kinematics can be controlled by the interaction between the articulating components and can also be affected by the different design parameters of the TKR. In force-controlled conditions, the AP and IE are driven from the applied force and the interaction between the articulating surfaces and do not, therefore, require a pre-knowledge of the AP and IE displacements. However, the force-controlled conditions are associated with more variables, as the effect of ligaments and muscles on kinematics, and are therefore difficult to be implemented. International standards for the wear simulation of TKRs have been developed for force and displacement control regimes, ISO 14243-1^9^ and ISO 14243-3,^8^ respectively. The appropriate control regime can therefore be selected depending on the level of intrinsic constraint of the knee replacement and on the research question. However, the resultant motion in force control is dependent on the dynamic and resistive characteristics of the simulator stations, as well as the choice of soft tissue–equivalent constraints and prosthesis design. The dependencies can produce high variability in the kinematics in different stations.

In order to simulate a wider range of physiological activities observed in different patient populations, such as younger more active patients, preclinical knee simulators with greater capabilities are required. A new six-station electromechanically driven knee simulator (Simulation Solutions, UK) with five fully independently controlled axes of articulation for each station, capable of replicating deep knee flexion as well as other adverse conditions experienced by young and more active patients, has been developed. The new knee simulator can be operated in either force or displacement control. This simulator has improved capability and can therefore fully meet and be capable of going well beyond the existing requirements of the relevant international standards.

This study investigated the wear of a fixed-bearing TKR using this second-generation electromechanically driven fully independent knee simulator and compared it to previous data from a first-generation predominantly pneumatically controlled simulator (ProSim knee simulator) that was not fully independently controlled. In addition, the kinematic performance and the repeatability of the simulator have been investigated and compared to the international standard requirements.^8^

## Materials and methods

Six Sigma fixed-bearing cruciate retaining TKRs (DePuy Synthes) comprising Co-Cr-Mo alloy femoral components, and polished Co-Cr-Mo tibial trays, were used with curved polyethylene tibial inserts. The inserts were moderately cross-linked (5 MRad irradiated and re-melted) GUR1020 ultra-high-molecular-weight polyethylene (UHMWPE; XLK™). The six sets of bearings were mounted anatomically in each station. The central axis of each implant was offset from the aligned axes of applied load and tibial rotation from the centre of the joint by 7% of its width, in accordance with the ISO recommendation.^8,9^ The centre of rotation of the femoral components was taken as the distal radius of the implant, as indicated by the device design.

**Figure 1.**
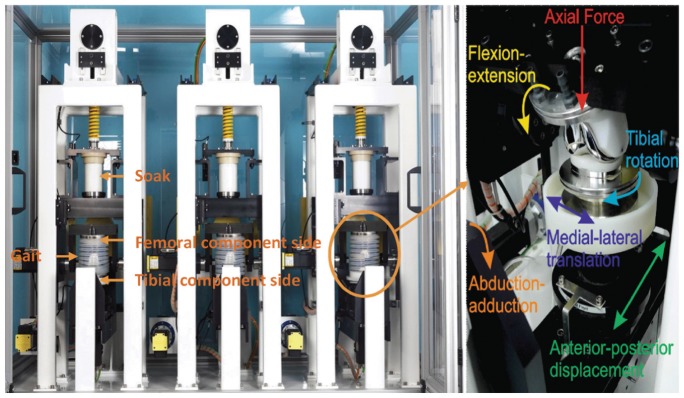
Six-station electromechanically driven knee simulator (Simulation Solutions, UK) and the controlled axes of articulation for each station.

The experimental simulation was run using the second-generation electromechanically driven fully independent knee simulator. The simulator had six fully independent stations in two banks; three stations each bank (Figure 1). Each station had 6 degrees of freedom with five controlled axes of motion – axial load to the femoral component, femoral flexion extension, tibial internal/external rotation, tibial AP displacement, and tibial adduction–abduction rotation. The simulator also allowed the ML motion to be predefined prior to test (Figure 1). In addition, the simulator has capacity for six loaded soak controls, not used in this study (Figure 1).

The second-generation knee simulator can run using either force- or displacement-controlled inputs with an operating frequency of up to 2 Hz. The simulator can apply up to ±25 mm AP tibial motion and ±25° IE tibial rotation. The simulator’s specifications are summarised and compared to the first-generation pneumatic simulator in Table 1. A six-axis load cell and displacement sensors on every controlled axis and station allowed the full range of output loading and kinematic profiles from the second-generation simulator to be obtained and compared to the demand input profiles.

**Table 1.** Specifications of second-generation electromechanical and first-generation pneumatic knee simulators

	Electromechanical knee simulator	Pneumatic knee simulator^25^
Fully controllable independent axes	Five	Three (FE-linked axis)
Operating frequency	Up to 2.0 Hz	Up to 2.0 Hz
Flexion–extension	Up to ±90°	Up to ±90°
Anterior–posterior	Up to ±25 mm	Up to ±13 mm
Internal–external	Up to ±25°	Up to ±10°
Adduction–abduction	Up to ±10° fully controllable or passive	Up to ±10° passive
Medial lateral	Up to ±10 mm pre-defined/fixed	Up to ±10 mm pre-defined/fixed
Axial loading	Up to 5 kN	Up to 5 kN

**Figure 2.**
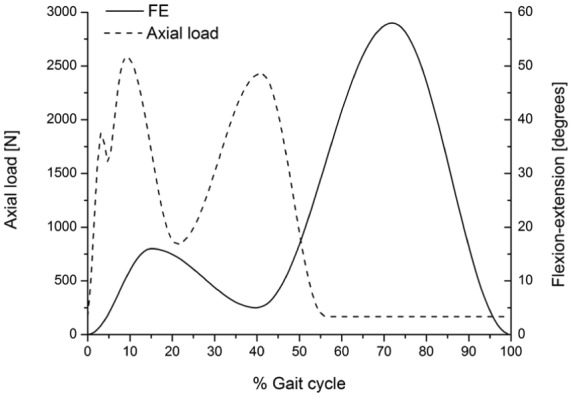
Flexion–extension (FE) and axial load input profiles.

**Figure 3.**
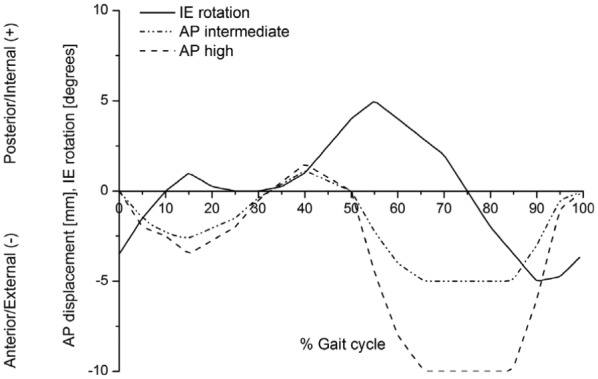
Internal–external rotation and anterior–posterior displacement input profiles.

Two test conditions were explored through the study. The femoral axis loading (maximum 2600 N) and flexion-extension (0°–58°) input profiles were taken from ISO 14243-3^8^ for both test conditions (Figure 2). The IE tibial rotation was displacement controlled and set at ±5° based on the natural kinematics of the knee as described by Lafortune et al.^36^ AP translation was displacement controlled, as this design of fixed-bearing knee replacement had minimal constraint and thus relies on soft tissue in vivo. The displacement test conditions used were intermediate kinematics with an AP displacement of maximum 5 mm and high kinematics with an AP displacement of maximum 10 mm^17^ (Figure 3). The femoral distal radius was taken as the femoral centre of rotation with a polarity of anterior tibial shift (denoted as negative AP motion) that produced femoral rollback. AA was allowed but not controlled. The inputs replicate conditions previously used over the last 15 years for the pneumatically controlled simulator.

The study was run for 3 MC each of high and intermediate kinematic conditions while maintaining the same TKRs in the same stations. At the end of this 6 MC period, the test was continued for a further 2 MC under high kinematics with the TKRs moved between stations to determine inter-station variability. The simulator was run at a frequency of 1 Hz. The lubricant used was new-born calf serum, diluted to 25%, supplemented with 0.03% (v/v) sodium azide to retard bacterial growth, and was changed every 0.33 MC. Prior to testing, all inserts were soaked in deionised water for a minimum period of 4 weeks. This allowed an equilibrated fluid absorption level to be achieved prior to the commencement of the wear study, reducing variability due to fluid weight gain. Wear was determined gravimetrically at 1 MC measurement intervals throughout the study. A Mettler XP205 (Mettler-Toledo, USA) digital microbalance, which had a readability of 0.01 mg, was used for weighing the bearing inserts. The volumetric wear (mean ± 95% CIs) was calculated from the weight loss measurements, using a density of 0.93 mg/mm^3^ for the polyethylene material and using unloaded soak controls to compensate for moisture uptake. The cumulative volumetric wear was calculated for each station and the mean wear rate was then calculated for all six stations, of each high and intermediate kinematics. The wear results were compared to previous data obtained for the same type of TKR under the same test conditions in a previous (first) generation (predominantly pneumatically controlled) simulator.^15^

The output kinematics from the pneumatic and the electromechanical knee simulators were recorded for the same type of TKR under the same test conditions. The output kinematics for each station were taken as the average kinematics of 25 different cycles throughout the test. These kinematics were then averaged for the six stations. In addition, the differences between the output and the input kinematics were calculated as a percentage of the corresponding maximum demanded value for the 25 cycles. These differences were then averaged for the six stations and compared to the ISO standard maximum tolerance of the output kinematics.^8,9^

Statistical analysis of the data was performed by first testing for homogeneity of variances and the means compared using the one-way analysis of variance (ANOVA) with 95% CI, and significance was taken at p < 0.05. This statistical analysis was performed using SPSS statistics (IBM SPSS, ver. 22, Armonk, NY, USA).

## Results

**Figure 4.**
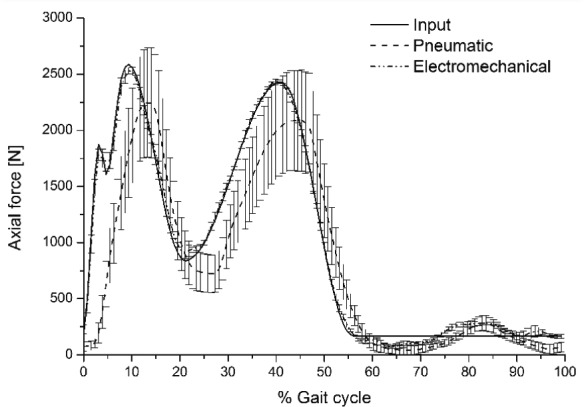
Input and average output (mean ± 95% CI, n = 6) axial load (N) from the pneumatic and electromechanical knee simulators.

The kinematic performance of the second-generation electromechanically driven knee simulator was investigated using PFC Sigma TKR (DePuy Synthes) and compared to that of the first-generation predominantly pneumatically controlled knee simulator. The second-generation knee simulator achieved the input maximum axial load and initial peak at heel strike. The maximum output axial loads at peaks were 1803 ± 52, 2530 ± 30, and 2435 ± 16 N (mean ± 95% CI, n = 6) compared to maximum input of 1875, 2589, and 2430 N, respectively (Figure 4). The maximum output axial loads at peaks from the first generation were 2248 ± 487 N and 2088 ± 452 N (mean ± 95% CI, n = 6). The first-generation knee simulator did not, however, achieve the initial peak at heel strike. In addition, the second-generation knee simulator achieved the input axial load to a maximum value of ±4.3% of the maximum input value, compared to a maximum value of ±64.8% of the maximum input value from the first-generation simulator. The ISO standard maximum tolerance is ±5% of the maximum input value.^8^

**Figure 5.**
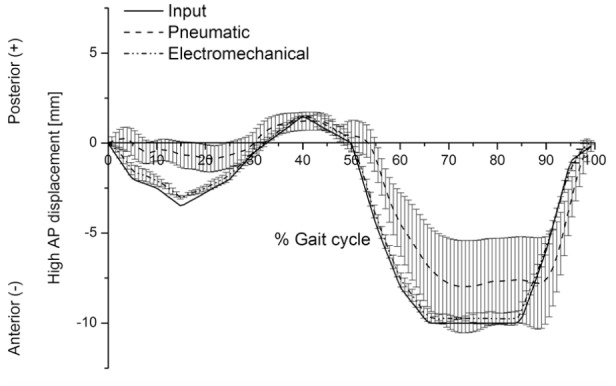
Input and average output (mean ± 95% CI, n = 6) anterior–posterior displacement (mm; high kinematics) from the pneumatic and electromechanical knee simulators.

The maximum output AP displacements under high kinematic conditions from the second-generation knee simulator were 3.0 ± 0.1 and 9.8 ± 0.4 mm (mean ± 95% CI, n = 6) during the stance and the swing phases, respectively. The maximum input AP displacements under high kinematics were 3.5 and 10.0 mm during the stance and the swing phases, respectively (Figure 5). The corresponding maximum average AP displacements achieved from the first-generation knee simulator were 1.2 ± 0.5 and 8.0 ± 2.6 mm (mean ± 95% CI, n = 6) during the stance and swing phases, respectively. The input AP displacement was achieved to a maximum value of ±4.7% and ±36.1% of the maximum input displacement for the second-generation and the first-generation knee simulators, respectively. The profile followed under intermediate kinematics was similar to that measured under high kinematics.

**Figure 6.**
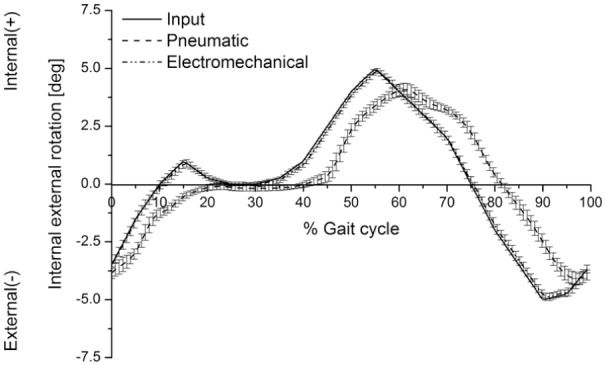
Input and average output (mean ± 95% CI, n = 6) internal–external rotation angle (°) from the pneumatic and electromechanical knee simulators.

The input IE rotation angle was achieved to a maximum value of ±4.5% and ±52.4% of the maximum input angle from the second-generation and the first-generation simulators, respectively. The maximum output values for the IE rotation angle were ±4.9° ± 0.2° and ± 4.1° ± 0.3° (mean ± 95% CI, n = 6) from the second-generation and the first-generation simulators, respectively. The maximum input values for the IE rotation angle were ±5° (Figure 6).

**Figure 7.**
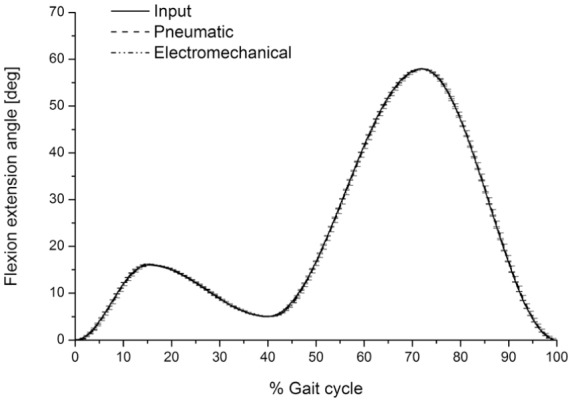
Input and average output (mean ± 95% CI, n = 6) flexion–extension angle (°) from the pneumatic and electromechanical knee simulators.

The second-generation and the first-generation knee simulators were both capable of accurately following the input FE rotation angle. The maximum output FE rotation angles were 16.0° ± 0.04° and 57.9° ± 0.03° (mean ± 95% CI, n = 6) during the stance and swing phases, respectively. The corresponding maximum input FE angles were 16° and 58° during the stance and swing phases, respectively. The input FE angle was achieved to a maximum value of ±1% of the maximum input values (Figure 7).

**Figure 8.**
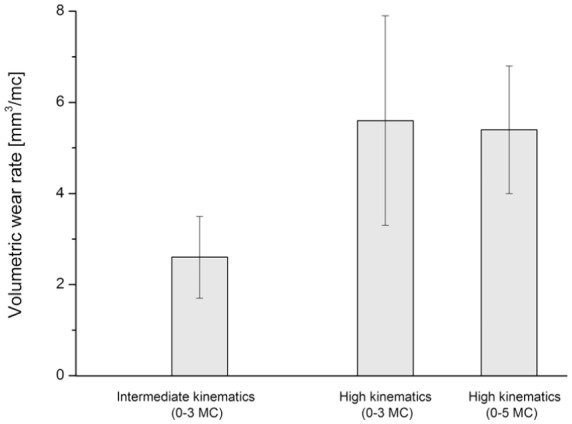
Volumetric wear rates under intermediate and high kinematic conditions (mean ± 95% CI, n = 6).

The wear increased linearly as the number of cycles increased. After 3 MC of intermediate and high kinematic conditions, the second-generation knee simulator produced mean wear rates of 2.6 ± 0.9 and 5.6 ± 2.3 mm^3^/MC (mean ± 95% CI, n = 6), respectively. After a further 2 MC of high kinematic conditions with the TKRs moved into adjacent stations every MC the mean wear rate was 5.4 ± 1.4 mm^3^/MC (mean ± 95% CI, n = 6; Figure 8).

**Figure 9.**
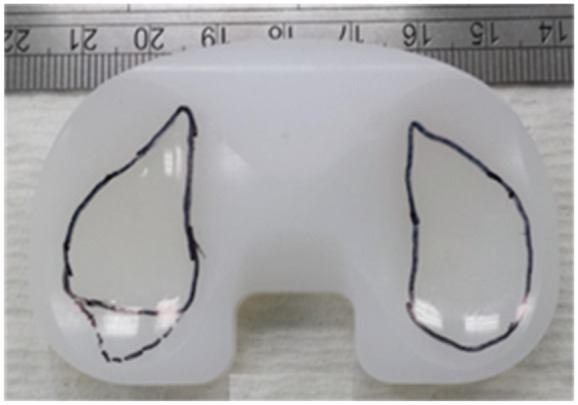
Wear scars for the Sigma fixed-bearing TKR after 3 MC of high kinematic conditions.

The wear scar from the second-generation knee simulator, after 3 MC of high kinematic inputs, is shown in Figure 9. Different stations showed similar wear scars. The mean wear scar area (percentage of total surface area) with 95% confidence limits for the high-kinematics test condition was 28.0% ± 3.0% (mean ± 95% CI, n = 6).

## Discussion

This study investigated the performance of a new generation of electromechanically driven fully independent simulator for assessment of TKR wear. The new-generation knee simulator was used to study the wear performance of the Sigma fixed-bearing cruciate retaining TKR (DePuy Synthes), with moderately cross-linked GUR1020 UHMWPE inserts, under two different levels of standard kinematic conditions (intermediate and high kinematics). The kinematic performance of the new-generation simulator was evaluated against the international standard requirements^8^ and the first-generation predominantly pneumatically controlled simulator (ProSim knee simulator) that was not fully independently controlled when running the same design of TKRs.

The actual delivered loading and kinematic profiles followed the input loading and kinematic profiles more closely on the second-generation simulator compared to the first generation. For example, the first-generation simulator did not achieve the initial peak on heel strike, and the maximum AP displacements and IE rotation were lower than those achieved by the second-generation simulator (Figures 4–7). The results from the first-generation pneumatic simulation were consistent with those of Barnett et al.^25^

In order to meet the international standard requirements for knee simulators, the control system of the knee simulator should be capable of generating loading cycles following the cycles given in Figures 2 and 3 and maintaining the magnitudes of these loading cycles to a tolerance of ±5% of the corresponding maximum demand value.^8^ The second-generation knee simulator could therefore meet the international standard requirements for the kinematics delivery under the standard loading conditions, with maximum deviations of less than ±5% of the corresponding maximum demand values. The first-generation pneumatic knee simulator could meet the international standard requirements for FE cycle since this axis was motor driven, with a maximum deviation of less than ±5% of the corresponding maximum demand angle. The maximum tolerances for axial load, AP, and IE cycles from the pneumatic simulator were, however, higher than ±5% of the corresponding maximum demand values. The pneumatic knee simulator could not therefore meet the standard requirements for knee simulators.^8^ In addition, due to the pneumatic control system of the simulator, the simulator could not rapidly respond to the axial load loading cycle which resulted in a lag in following the axial load profile. (Figure 4).

Despite the closer following of the input profiles from the second-generation knee simulator, inter-station variability in terms of varying wear rates still existed in the second-generation simulator (Figure 8). This inter-station variability suggests that other factors such as differences in alignment of the TKRs, station geometry and station set up played a role. There is no recommendation in knee simulator wear testing as to whether to move the implants from one station to another periodically or not throughout the wear test. In this study, however, in order to account for the inter-station variability, the test was continued for a further 2 MC under high kinematics and after the 6 MC period with the TKRs moved between stations. The results showed that moving the TKRs between the stations reduced the effect of inter-station variability on wear. The measured average wear rate did not, however, significantly change (p = 0.99; Figure 8).

**Figure 10.**
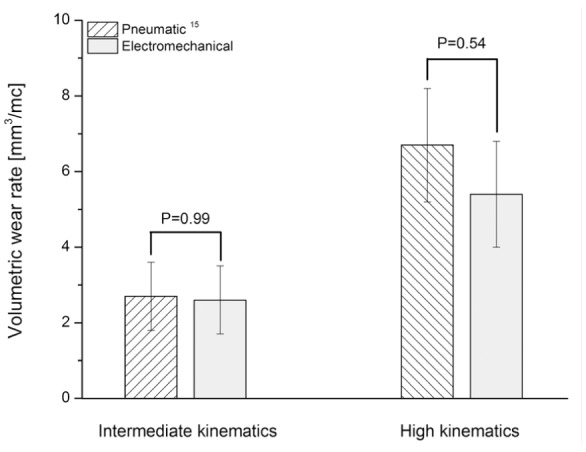
Volumetric wear rates (mean ± 95% CI, n = 6) under intermediate and high kinematic conditions measured from pneumatic^15^ and electromechanical knee simulators.

**Figure 11.**
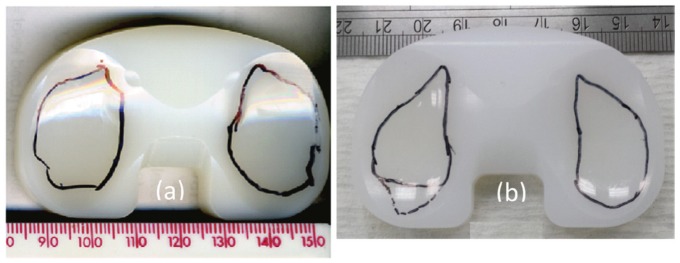
Wear scars for the Sigma fixed-bearing TKR after 3 MC of high kinematic conditions in (a) pneumatic^14^ and (b) electromechanical knee simulators.

The wear rates from the second-generation knee simulator were not significantly different from those reported from the first-generation knee simulator^15^ (Figure 10). The measured wear rates from the same TKR design and for the same material using the first-generation pneumatic knee simulator were 2.7 ± 0.9 mm^3^/MC (p = 0.99) and 6.7 ± 1.5 mm^3^/MC (p = 0.54) under intermediate and high kinematics, respectively.^15^ The reported mean wear scar area for the high-kinematics test condition from the first-generation knee simulator (34.0 ± 6.0 (mean ± 95% CI, n = 6)^14^) was, however, higher than that measured in this study from the second-generation knee simulators under high kinematic conditions (28.0 ± 3.0% (mean ± 95% CI, n = 6); Figure 11). The differences in wear scars were attributed to the differences between the studies, such as different knee simulators and TKR components.

This study used displacement control input profiles to validate the simulator, rather than force-controlled profiles. The rationale for this was to enable comparison with previous data and to reduce the range of variables (i.e. force control can produce a greater range of displacements, which is not helpful in simulator validation).

Future work will undertake parallel studies under force as well as displacement control input conditions to evaluate a greater range of variables that can affect the reliability and performance of knee replacements in line with our Stratified Approach For Enhanced Reliability (SAFER^®^) approach.^24^ Such variables will include, for example, the surgical positioning of the components and range and type of activities. The improved capability and performance of the second-generation electromechanically driven knee simulators make them ideal to accurately simulate such variations, which may be responsible for the higher failure rate of TKR in younger and more active patients. These advances in experimental simulation will be combined with advances in computational simulation^37^ for an optimal simulation and wear prediction approach. In this approach, experimental simulation is first used to run a limited set of variations, required for the validation of the computational simulation. The computational simulation can then be used to investigate a much wider range of clinically relevant conditions as well as parametric wear and kinematic simulation studies. The combined experimental–computational simulation approach can therefore be a significant simulation tool for further improvement of the reliability and performance of the TKR implant system.

## Conclusion

The wear rates from the electromechanical and pneumatic knee simulators were not significantly different. However, the output kinematic profiles followed the input kinematic profiles more closely on the electromechanical simulator than the pneumatic simulator. In addition, the electromechanical simulator was capable of following kinematic and loading input cycles within the tolerances of the international standard requirements.^8^ The new-generation knee simulator with fully independent control can therefore be used for a much wider range of kinematic conditions, including high-flexion and other demanding conditions, due to its improved capability and performance over the pneumatic simulators, which will be the focus of future studies.
